# Study on Mechanical Strength and Resistance to Freeze–Thaw Cycles and Chloride Ions of Fly Ash Mortar Mixed with Limestone Powder Cured Under Low Temperature

**DOI:** 10.3390/ma18204814

**Published:** 2025-10-21

**Authors:** Qingfeng Chen, Weizhun Jin, Jingjing Li, Min Huang, Pengfei Fang, Yuru Zhao

**Affiliations:** 1 College of Civil Engineering, Henan University of Engineering, Zhengzhou 451191, China; chenqingfeng@haue.edu.cn (Q.C.); jianai729505@163.com (J.L.); huangmin_2006@163.com (M.H.); zhaozhao6680@163.com (Y.Z.); 2 College of Civil and Transportation Engineering, Hohai University, Nanjing 210024, China; 3 College of Civil Engineering, Henan University of Technology, Zhengzhou 450001, China; fangpf_2018@163.com

**Keywords:** limestone powder, fly ash, cement mortar, mechanical strength, durability

## Abstract

Fly ash (FA) and limestone powder (LS) are both environmentally friendly substitutes for cement. The mechanical strength and durability of hydraulic concrete with FA and LS cured under low temperature deserve attention. In this study, the mechanical strength and durability properties of cement mortar based on FA and LS cured under a low temperature of 5 °C were investigated. The compressive strength and resistance to freeze–thaw cycles and chloride ions of mortar with FA and LS were evaluated. The results reveal that adding 5% LS can increase the compressive strength of mortar containing 30% FA by 14.31% at 3 d and 1.63% at 180 d. The addition of 5% LS can result in a 0.58% reduction in the mass loss rate and an increase of 2.98% in RDM compared to the mortar with 30% FA after 100 freeze–thaw cycles. Meanwhile, the addition of 5% LS results in a 5.68% reduction in the chloride diffusion coefficient compared to the mortar with 30% FA. However, for the mortar containing 60% FA, any proportion of LS cannot increase the compressive strength of FA mortar from 3 d to 180 d and will decrease the resistance to freeze–thaw cycles and chloride ions. For the mortar containing 30% FA, the addition of 5% LS can promote the hydration product of C-S-H and obtain the minimum total amount of large capillary pores and air pores. Adding 10% LS can result in the mortar with 30% FA obtaining the lowest unit CO_2_ emission, and adding 5% LS can result in the mortar with 60% FA obtaining the lowest unit CO_2_ emission.

## 1. Introduction

In modern construction projects, concrete is still the most widely used artificial building material, and its usage is still increasing rapidly [1,2,3]. However, the production of cement, the main cementitious material in concrete, is an unsustainable industry with high energy and resource consumption as well as high pollution. Cement production consumes a large amount of non-renewable energy, such as coal and natural gas, and a large amount of non-renewable natural resources, such as limestone and clay. In addition, the process of cement production will emit a large amount of greenhouse effect of CO_2_, resulting in the global average temperature year by year [4,5]. Substituting a portion of cement with mineral admixtures not only reduces the amount of cement but also solves the pollution problem of industrial wastes [6,7]. Due to the excellent performance in concrete, fly ash (FA) has been widely concerned [8,9]. As a kind of active mineral admixture, FA plays the role of morphology effect, micro-aggregate effect, and pozzolanic effect in cement-based materials [10,11], which can reduce the internal friction resistance between particles and increase the fluidity, fill the large holes in cement stone to improve the pore structure of concrete, and react with Ca(OH)_2_ to generate new C-S-H and calcium aluminate hydrate to strengthen the concrete structure [12,13,14]. Hence, utilizing the high blending FA to replace part of cement in concrete is not only conducive to energy saving and environmental protection but also maintains the high performance of concrete. However, the slow hydration of FA results in insufficient early cementitious products, causing the internal structure of concrete to be loose, which not only leads to a significant drop in the early strength of concrete but also weakens the durability of concrete. Furthermore, in recent years, due to the expansion of construction scale, the demand for FA has increased sharply, resulting in a large shortage in some places. The long-distance transportation of FA is bound to bring a large economic burden. Therefore, a new mineral admixture is urgently needed to improve the deficiencies and resource shortage of FA.

Limestone powder (LS) is an inorganic, non-metallic powder material mainly composed of calcium carbonate (CaCO_3_). It is usually produced by processing natural limestone ores through processes such as crushing and grinding. Limestone is one of the most widely distributed minerals on Earth, so the LS resources are extremely abundant and readily available. LS, due to its excellent performance in enhancing the properties of concrete and its low-carbon and environmentally friendly features, has been increasingly applied in construction projects [15,16,17]. LS can reduce the carbon footprint of concrete by approximately 15% and also decrease the raw materials required for cement production by about 10% [18]. The particle size of LS is usually smaller than that of cement particles, which can fill the gaps between cement particles and form a more compact stacking structure [19,20]. LS can act as a nucleation agent for C-S-H gel and Ca(OH)_2_, promoting the orderly growth of hydration products, reducing the formation of amorphous gels, and making the structure denser [21,22]. The CaCO_3_ in LS reacts with the cement hydration products Ca(OH)_2_ and tricalcium aluminate (C_3_A), generating calcium carboaluminate that fills the structural pores and provides strength [23,24]. Therefore, LS can not only solve the problem of low early strength of FA concrete but also reduce the engineering cost and provide a feasible path for the low-carbon and resource-based development of the building materials industry. Furthermore, when concrete structures are constructed in high latitude and cold areas, the concrete often needs to be exposed to low temperatures in the hardening stage, and the mechanical strength of concrete at the early hardening stage and durability at the later stage need to be paid attention [25,26]. Meanwhile, hydraulic concrete is often damaged by the actions of freeze–thaw cycles and chloride ion erosion. The foundations of coastal buildings, cross-sea bridges, and underwater tunnels, etc., will all be subject to the erosion of chloride ions. The seawater seeps through the concrete pores, and chloride ions destroy the passivation film on the surface of reinforcing bars, causing electrochemical corrosion [27,28]. The expansion of rust leads to the cracking and peeling of concrete. Meanwhile, buildings in the northern high-dimensional and cold regions, as well as engineering projects in high-altitude mountainous areas, will all suffer severe damage from the repeated freeze–thaw cycles [29,30]. The water in the internal pores of concrete freezes at low temperatures (expanding by approximately 9% in volume), exerting mechanical pressure on the pore walls; when it melts, the pressure is released, and the pore structure gradually deteriorates. After multiple cycles, the concrete develops microcracks and flaking, and its strength significantly decreases. Based on the above analysis, it can be concluded that low-temperature curing significantly inhibits the hydration process of cement in concrete, resulting in a loose microstructure and increased porosity. This, in turn, exacerbates the risks of freeze–thaw cycles and chloride ion erosion. LS, through the physical filling—chemical synergy—structural regulation triple mechanism, can improve the defects of low-temperature curing and significantly enhance the concrete’s resistance to freeze–thaw cycles and resistance to chloride ion erosion. Meanwhile, research on the combined effect of FA and LS on the resistance to freeze–thaw cycles and chloride ions of concrete under low curing temperature conditions is currently lacking. Hence, a study on the resistance to chloride ion erosion and freeze–thaw cycles of concrete mixed with FA and LS cured under low temperature has important guiding significance for the application of FA and LS in hydraulic concrete cured under low temperature.

In this study, the mechanical strength and durability properties of cement mortar based on LS and high blending FA cured under low temperature were investigated. The compressive strength and resistance to freeze–thaw cycles and chloride ions of mortar with FA and LS were evaluated. The hydration products and microstructure of mortar were characterized by XRD, TG, SEM, and MIP tests.

## 2. Experimental Procedure

### 2.1. Materials

**Figure 1:**
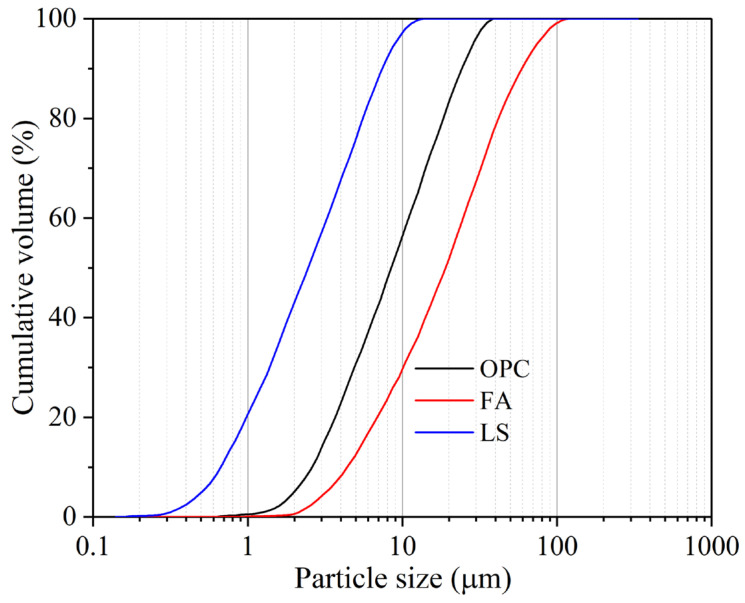
Particle size distributions of OPC, FA, and LS.

In this study, the materials used include P•O 42.5 ordinary Portland cement (OPC), FA, and LS with specific surface areas of 327 m^2^/kg, 253 m^2^/kg, and 741 m^2^/kg, respectively. The densities of OPC, FA, and LS are 3.06 g/cm^3^, 2.18 g/cm^3^, and 2.72 g/cm^3^, respectively. The particle size distributions of these three raw materials are shown in Figure 1. The distribution of LS in the smaller particle size range is much larger than that of OPC and FA, indicating that the particle size of LS is much smaller than that of OPC and FA. Meanwhile, FA has the widest distribution in the larger particle size range, indicating that FA has a much larger particle size than OPC and LS. Table 1 lists the chemical compositions of OPC, FA, and LS. The aggregate is the China ISO standard sand. Superplasticizer (SP) is a high-performance polycarboxylic acid purchased from Sobute New Materials Co., Ltd. (Nanjing, China).

### 2.2. Specimen Preparation

Table 2 displays the mix proportions of mortar. In order to achieve a high early strength for FA mortar under low-temperature curing conditions and to ensure complete hydration of FA mortar, the water-to-binder ratio is set as 0.35. Based on the commonly used dosages of FA in actual engineering projects, the replacement amount of FA for cement is set as 30% and 60%, respectively. According to previous scholars’ research, the addition of LS within 20% is considered beneficial for the mechanical and durability properties of cement-based materials. Therefore, the replacement amount of LS is set as 5%, 10%, and 15%, respectively. In order to improve the fluidity of mortar, 0.5% SP by mass of binder is added into each proportion. The mortar was poured into the 40 mm × 40 mm × 40 mm molds to test compressive strength and conduct resistance to freeze–thaw cycles. Meanwhile, the mortar was poured into Φ100 mm × 50 mm cylindrical molds to test the chloride diffusion coefficient. All mortar blocks were cured at 5 °C under a water bath.

### 2.3. Test Methods

#### 2.3.1. Compressive Strength

The compressive strength was measured according to Chinese standard GB/T 17671-2021 [31]. The compressive strength was determined by using cement mortar blocks, and the load was uniformly added at a rate of 2400 N/s until broken. The average compressive strength of three samples was ultimately calculated as the result.

#### 2.3.2. Freeze–Thaw Resistance

After the mortar blocks were cured for 180 d, the freeze–thaw resistance test was conducted according to Chinese standard GB/T 50082-2009 [32]. The compressive strength and mass loss of mortar were tested every 25 freeze–thaw cycles. The mass loss rate was obtained by Equation (1).

(1) $$W_{n}=\frac{M_{0}-M_{n}}{M_{0}}\times 100\%$$

where $W_{n}$ is the mass loss rate; $M_{0}$ is the mass before freeze–thaw cycles; $M_{n}$ is the mass after n freeze–thaw cycles.

Ultrasonic testing can determine the degree of damage to the sample after undergoing freeze–thaw cycles. When cracks occur within the mortar, it will cause an extension in the transmission time of ultrasonic waves within the sample. Therefore, by detecting the transmission time of ultrasonic waves, the changes in cracks and defects within the mortar can be determined, that is, the degree of freeze–thaw damage. In this experiment, a C62 non-metallic ultrasonic testing instrument was used to measure the ultrasonic propagation time after different freeze–thaw cycles. The relative dynamic elastic modulus (RDM) is calculated using the following Equations (2) and (3).

(2) $$DM=\frac{(1+v)(1-2v)\rho V^{2}}{1-v}=\frac{(1+v)(1-2v)\rho L^{2}}{(1-v)t^{2}}$$

(3) $$RDM=\frac{E_{dn}}{E_{d0}}=\frac{V_{n}^{2}}{V_{0}^{2}}=\frac{t_{0}^{2}}{t_{n}^{2}}$$

where DM is dynamic elastic modulus; RDM is relative dynamic elastic modulus; V is propagation speed of ultrasonic waves within the mortar, m/s; L is the length of mortar block, m; ρ is the density of mortar; v is Poisson’s ratio; $t_{0}$ is the ultrasonic wave propagation time before the mortar undergoes freeze–thaw cycles, μs; $t_{n}$ is the ultrasonic wave propagation time after the mortar undergoes n freeze–thaw cycles, μs.

#### 2.3.3. Chloride Diffusion Coefficient

After mortar blocks were cured for 180 d, the chloride diffusion coefficient test was conducted by the RCM method corresponding to Chinese standard GB/T 50082-2009 [32]. The chloride diffusion coefficient is calculated according to Equation (4).

(4) $$D_{RCM}=\frac{0.0239\times (273+T)L}{(U-2)t}\left(X_{d}-0.0238\sqrt{\frac{(273+T)LX_{d}}{U-2}}\right)$$

where $D_{RCM}$ is the chloride diffusion coefficient; U is the absolute value of voltage, V; T is the average of the initial and ending temperatures of anode solution, °C; L is the thickness of the test block, mm; $X_{d}$ is the average penetration depth of chloride ions, mm; t is the duration of the test, h.

#### 2.3.4. Characterizations

The hydration products of the samples were characterized by XRD and TG. The cement mortar was dried at 50 °C for 24 h and broken up into powders. The XRD patterns were achieved using Bruker D8 Advance (Bruker AXS, Karlsruhe, Germany) under 40 kV and 40 mA from 5° to 60° at a speed of 10°/min. A TG test was conducted by STA449F5 Jupiter (NETZSCH Group, Selb, Germany), and the powders of mortars were heated from 30 °C to 900 °C at a speed of 10 °C/min.

The micromorphology and microporous structure of mortars were characterized by SEM and MIP. The sample was broken up into small blocks and dried at 50 °C for 24 h. Finally, the blocks were coated with gold, and SEM images were obtained using Zeiss Sigma 300 (CARL ZEISS, Oberkochen, Germany). The MIP test was conducted by AutoPore 9500 (Micromeritics Instrument Corporation, Norcross, GA, USA), and the samples were the same blocks as that in the SEM test.

#### 2.3.5. Environmental Evaluation

In order to better assess the environmental impact of FA and LS replacement cements, Equation (5), for evaluating environmental effects, was adopted [33]. The $embodied-CO_{2}$ index (CI) of the unit mortar compressive strength can reflect the relationship between CO_2_ emissions and compressive strength of mortar containing FA and LS.

(5) $$CI=\frac{embodied-CO_{2}}{f_{c}}$$

where $embodied-CO_{2}$ can be abbreviated as $e-CO_{2}$, kg/m^3^; $f_{c}$ is the compressive strength, MPa. In concrete construction, the emissions of CO_2_ mainly come from the production of raw materials, transportation, and the curing process. Due to the abundant and readily available resources of FA and LS, it is assumed that their transportation distances are relatively short. Therefore, the CO_2_ emissions generated during transportation can be disregarded. Furthermore, since the low-temperature curing process takes into account the low-temperature environment that the concrete is exposed to, which is determined by the climate, the CO_2_ emissions during the curing process can also be disregarded. Therefore, the CO_2_ emissions released during the production of the raw materials for the mortar constitute the total CO_2_ emissions.

The total $e-CO_{2}$ per unit volume in the mortar is obtained by adding up the $e-CO_{2}$ of each component. The $e-CO_{2}$ emissions from cement, FA, and LS in the mortar have been evaluated by previous studies, as shown in Table 3.

## 3. Results and Discussion

### 3.1. Compressive Strength

**Figure 2:**
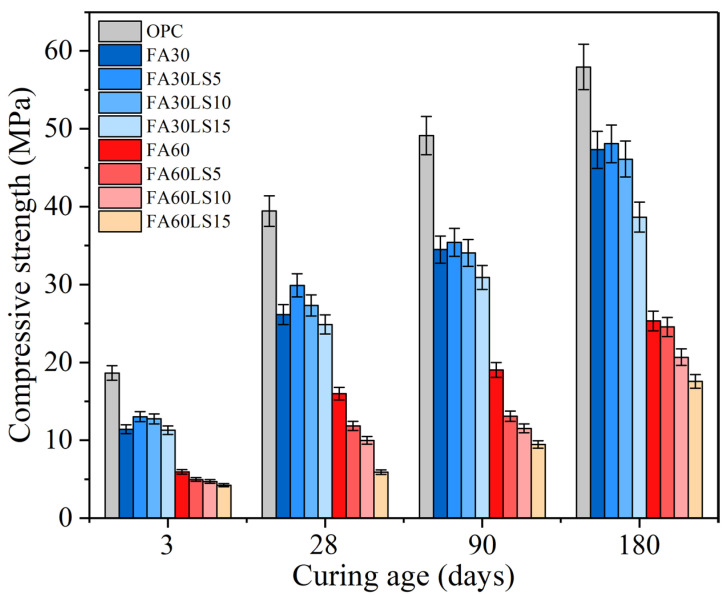
Compressive strength of FA mortars with different dosages of LS.

Figure 2 displays the compressive strength of FA mortars with different dosages of LS. It can be clearly observed that the compressive strength of FA mortars with different dosages of LS is much lower than that of the mortars without FA and LS at each curing age. This is mainly due to that, at a low temperature of 5 °C, the hydration rate of C_3_S and C_2_S in cement will be significantly slowed down, resulting in insufficient generation of CH and a slow generation rate. This directly deprives the pozzolanic reaction of FA of the necessary raw materials [39]. Meanwhile, the low temperature of 5 °C will significantly reduce the dissolution rate of the active components SiO_2_ and Al_2_O_3_ in FA and also slow down the nucleation and growth rate of C-S-H, resulting in the fact that, even at 90 d or 180 d, the activity of FA has not been fully stimulated and cannot provide sufficient strength. Furthermore, when 30% FA is added, a dilution effect occurs, which directly leads to a decrease in the total amount of C-S-H and CH that can be hydrated. Therefore, in the mortar with 30% FA added, the cement is diluted, and the pozzolanic reaction of FA is inhibited by low temperature. It is unable to make up for the gap caused by the reduction in cement usage by generating additional C-S-H, resulting in the compressive strength being even lower than that of the mortar without FA addition at 90 d and 180 d. Furthermore, the addition of small amounts of LS cannot significantly improve the strength of FA mortar. This is because 5% LS will further reduce the amount of cement used, resulting in a decrease in the total amount of C-S-H formed during the cement hydration process. The less generated C-S-H cannot fully encapsulate the LS and FA particles, resulting in interface voids still existing between the particles, and the micro-aggregate effect of LS cannot be fully exerted. Meanwhile, under a low curing temperature of 5 °C, the reaction rate between CaCO_3_ from LS and C_3_A is extremely slow. Therefore, within 90 to 180 d, very little calcium carboaluminate is formed, and it is almost impossible to compensate for the loss of bonding strength. This phenomenon was also observed in Weerdt’s research [40], namely, that the compressive strength of FA cement paste under low curing temperature of 5 °C was significantly lower than that of the cement paste without FA at 90 d, and even with the addition of 5% LS, it was far from reaching the compressive strength of the cement paste without FA.

Furthermore, for the mortar containing 30% FA, the addition of LS has a different influence on the strength of the FA mortar. Both 5% and 10% LS can improve the compressive strength at 3 d and 28 d. However, at 90 d and 180 d, only 5% LS can improve the strength of FA mortar. Adding 5% LS can increase the compressive strength of FA mortar by 14.31% at 3 d and 1.63% at 180 d. Meanwhile, 15% LS will reduce the compressive strength from 3 d to 180 d. Adding 15% LS will reduce the compressive strength of FA mortar by 0.97% at 3 d and 18.30% at 180 d. The main reason for the above phenomenon is caused by the influence effects of LS. In the early stage of hydration, LS can play a nucleation effect and promote the rapid hydration of cement particles on its surface [23,41]. In addition, the small amount of LS can play a role in the compaction of the internal structure of FA mortar [42]. Furthermore, LS itself is chemically active and can react with C_3_A in cement to form calcium carboaluminate, thus strengthening FA mortar [43]. Therefore, 5% LS can play these three roles in FA mortar, which has a strengthening effect on FA mortar from 3 d to 180 d. For 10% LS, in the early curing stage from 3 d to 28 d, the three effects of LS enable it to strengthen FA mortar, but in the later stage from 90 d to 180 d, due to the weakening of the nucleation effect of LS, the negative effect of the reduction in hydration products caused by the replacement of cement by 10% LS gradually becomes prominent, making the strength of FA mortar gradually decrease. For a large amount of LS (15%), the strengthening effect of the three effects of LS on FA mortar is much smaller than the negative effect of LS on the reduction in hydration products caused by cement replacement, leading to a decrease in the strength of FA mortar from 3 d to 180 d.

For the mortar containing 60% LS, any proportion of LS cannot increase the compressive strength of FA mortar from 3 d to 180 d, and with an increase in LS dosages, the compressive strength continues to decrease. Adding 15% LS will reduce the compressive strength of FA mortar by 29.03% at 3 d and 30.62% at 180 d. This is mainly due to the fact that, in this system, 60% of the cement has already been replaced by FA, and further addition of LS will result in even less cement, resulting in very few hydration products with bonding power. Meanwhile, the enhancement effect brought by the nucleation effect, filling effect, and chemical activity effect of LS on FA mortar is far from making up for the adverse negative effect caused by the sharp reduction in hydration products caused by the reduction in cement. Therefore, LS cannot enhance the mortar containing 60% FA at any age.

By comparing the research results of this study with those of previous studies, it was found that this study has some new findings. In Weerdt’s research [40], 5% LS was found to effectively enhance the compressive strength of FA pastes under curing conditions of 5 °C and 20 °C at ages from 1 d to 90 d. However, in Sezer’s study [44], 5% LS led to a decrease in the compressive strength of cement pastes cured under 20 °C at ages from 2 d to 180 d. Furthermore, in Huang’s research [45], 10% LS was able to significantly increase the compressive strength of concrete without FA at 28 d, while causing a sharp decrease in the compressive strength of concrete with 30% FA at 28 d. Meanwhile, in our previous research [19], 10% LS led to a decrease in the compressive strength of concrete cured under 20 °C at ages from 3 d to 90 d. In this study, under a curing temperature of 5 °C, the addition of 5% and 10% LS can improve the compressive strength of mortar with 30% FA at 3 d and 28 d, and only 5% LS can improve the compressive strength of mortar with 30% FA at 90 d and 180 d. Meanwhile, any dosage of LS cannot increase the compressive strength of mortar with 60% FA at ages from 3 d to 180 d, and with an increase in the dosage of LS, the compressive strength of mortar with 60% FA decreases.

### 3.2. Compressive Strength After Freeze–Thaw Cycles

**Figure 3:**
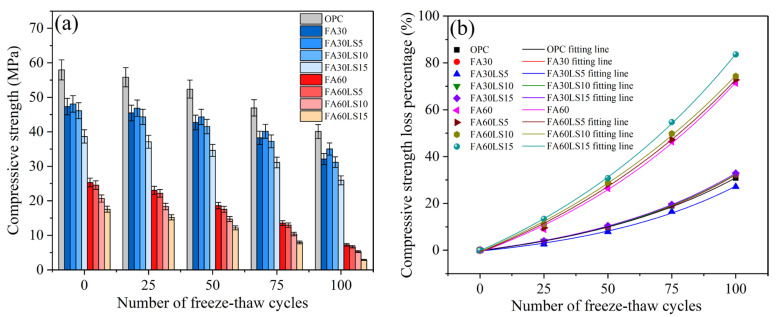
(**a**) Compressive strength and (**b**) compressive strength loss percentages of FA mortars containing different dosages of LS after freeze–thaw cycles.

Figure 3a displays the compressive strength of FA mortars containing different dosages of LS after 100 freeze–thaw cycles. The compressive strength of FA mortars decreases to varying degrees after 100 freeze–thaw cycles. Meanwhile, the compressive strength of FA mortars with different proportions still maintains the original law; that is, FA mortar with higher compressive strength still has higher compressive strength after freeze–thaw cycles.

Figure 3b displays the compressive strength loss percentages of FA mortars containing different dosages of LS. The compressive strength loss percentages of mortar with 60% FA are much greater than those of mortar with 30% FA. This indicates that the freeze–thaw resistance of mortar with 60% FA is much less than that of mortar with 30% FA. This is mainly because the pozzolanic activity of FA is inhibited when the amount of FA is too large, resulting in low freeze–thaw resistance. Furthermore, for the mortar containing 30% FA, the compressive strength loss percentages of FA mortar are significantly reduced by adding 5% LS, and it is much lower than that of the mortar without FA. The compressive strength loss percentages of FA mortar with 5% LS decrease by 4.98% and 3.66% compared with the mortar containing 30% FA and the mortar without FA and LS after 100 freeze–thaw cycles. This is because the filling effect and chemical activity effect of LS enhance the freeze–thaw resistance of FA mortar [21,46]. However, the addition of 10% and 15% LS will lead to compressive strength loss percentages of FA mortar that are slightly greater than those of FA mortar. The compressive strength loss percentages of FA mortar with 15% LS increase by 0.77% and 2.09% compared with the mortar containing 30% FA and the mortar without FA and LS after 100 freeze–thaw cycles. This is because the filling effect and chemical activity effect of LS on the enhancement of FA mortar’s freeze–thaw resistance is slightly smaller than the reduction in hydration products caused by LS replacing cement on the weakening of FA mortar’s freeze–thaw resistance. For the mortar containing 60% FA, the continuous addition of LS will gradually increase the compressive strength loss percentages of the FA mortar. The compressive strength loss percentages of FA mortar with 15% LS increase by 12.24% and 52.78% compared with the mortar containing 60% FA and the mortar without FA and LS after 100 freeze–thaw cycles. This is because the large amount of FA has led to a serious reduction in hydration products, and the continued addition of LS will further reduce the hydration products, which will seriously reduce the freezing resistance of mortar. Meanwhile, the filling effect and chemical activity effect of LS on the improvement of freeze–thaw resistance of mortar with very few hydration products caused by high dosage of FA are too small and can be ignored. Moreover, the compressive strength loss percentages of FA mortars increase with an increase in freeze–thaw cycles, and the change law of compressive strength loss percentages with freeze–thaw cycles conforms to Equation (6). Table 4 lists the correlation coefficients of fitting curves between compressive strength loss percentages and freeze–thaw cycles, and all R^2^ larger than 0.99 prove the accuracy of the fitting lines.

(6) σ=A∗exp(n/B)+C

### 3.3. Mass Loss Rate and RDM After Freeze–Thaw Cycles

**Figure 4:**
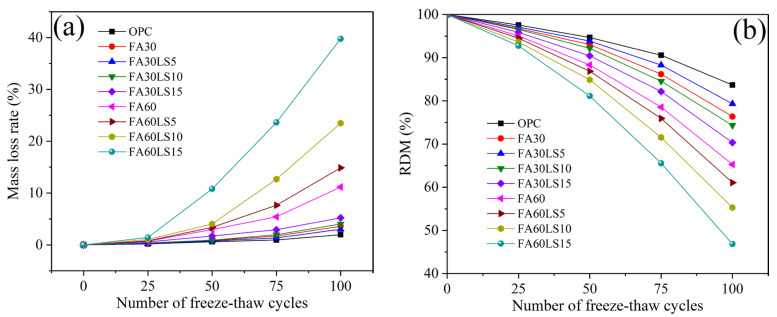
(**a**) Mass loss rates and (**b**) RDM of FA mortars containing different dosages of LS after freeze–thaw cycles.

Figure 4a and 4b present mass loss rates and RDM of FA mortars containing different dosages of LS, respectively. Whether LS is added or not, the mass loss rate of 30% FA mortar is much lower than that of 60% FA mortar, and its RDM is much higher than that of 30% FA mortar. This indicates that the freeze–thaw resistance of 30% FA mortar with or without LS is much higher than that of 60% FA mortar with or without LS. Furthermore, for the mortar containing 30% LS, after adding 5% LS, the mass loss rate of the FA mortar decreases, and the RDM increases. The addition of 5% LS results in a 0.58% reduction in the mass loss rate and an increase of 2.98% in RDM compared to the mortar with 30% FA after 100 freeze–thaw cycles. This indicates that 5% LS can improve the freeze–thaw resistance of FA mortar due to its filling effect and chemical activity effect in FA mortar [21]. However, after the addition of 10% and 15% LS, the mass loss rates of FA mortars increase, and the RDM decreases. The addition of 15% LS leads to an increase of 1.62% in the mass loss rate and a 6.04% reduction in RDM compared to the mortar with 30% FA after 100 freeze–thaw cycles. This indicates that 10% and 15% LS will result in a decrease in freeze–thaw resistance of FA mortar. This is due to the fact that the effect of excess LS on freeze–thaw resistance of FA mortar is less than that of LS on the decrease in the freeze–thaw resistance caused by the reduction in hydration products caused by cement replacement, leading to a slight decrease in overall freeze–thaw resistance of FA mortar. Moreover, for the mortar containing 60% LS, with the addition of LS increasing from 5% to 15%, the mass loss rate of FA mortar increases sharply, while RDM decreases sharply. The addition of 15% LS leads to an increase of 28.59% in the mass loss rate and an 18.41% reduction in RDM compared to the mortar with 60% FA after 100 freeze–thaw cycles. This indicates that the addition of LS will seriously reduce the freeze–thaw resistance of FA mortar in the mortar with large FA dosages. This is mainly because the replacement of cement by 60% FA severely reduces hydration products, and the replacement of cement by LS will further reduce hydration products, leading to a serious reduction in the freeze–thaw resistance of FA mortar.

### 3.4. Chloride Diffusion Coefficient

**Figure 5:**
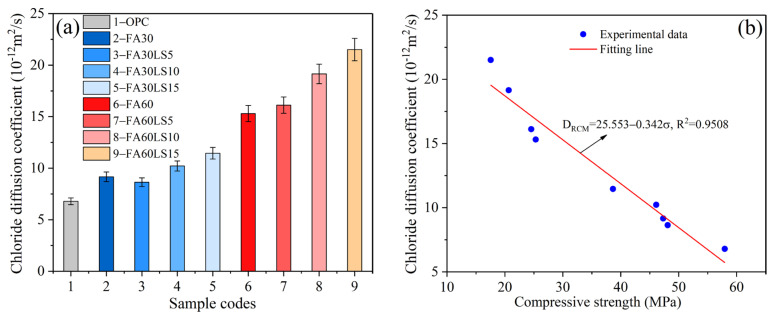
(**a**) Chloride diffusion coefficient of mortars; (**b**) relationship between chloride diffusion coefficient and compressive strength.

Figure 5a displays the chloride diffusion coefficient of FA mortars containing different dosages of LS. It can be seen that, for the FA mortars with or without LS, the chloride diffusion coefficient of 30% FA mortar is much lower than that of 60% FA mortar. This reveals that the chloride ion resistance of 30% FA mortar with or without LS is much higher than that of 60% FA mortar with or without LS. Moreover, for the mortar containing 30% LS, after adding 5% LS, the chloride diffusion coefficient of FA mortar decreases. The addition of 5% LS results in a 5.68% reduction in the chloride diffusion coefficient compared to the mortar with 30% FA. This demonstrates that less LS can improve the chloride ion erosion resistance of FA mortar. This is due to the filling effect of LS, which can be filled between the cement particles and the aggregate, thus compacting the FA mortar [19]. Meanwhile, the calcium carboaluminate produced by the chemical activity effect of LS can not only strengthen the FA mortar but also compact the microstructure of FA mortar. However, after the addition of 10% and 15% LS, the chloride diffusion coefficient of FA mortar increases a little compared with FA mortar without LS. The addition of 15% LS leads to an increase of 25.11% in the chloride diffusion coefficient compared to the mortar with 30% FA. This indicates that 10% and 15% LS will result in an increase in the chloride diffusion coefficient of FA mortar. This is due to that, when the replacement amount of LS to cement is large, the hydration products in FA mortar will be further reduced, resulting in the loose structure of FA mortar. Meanwhile, the filling effect and chemical activity effect of LS on the compaction of FA mortar cannot make up for the loose structure of FA mortar caused by the excessive replacement of cement by LS. For the mortar containing 60% LS, with the addition of LS increasing from 5% to 15%, the chloride diffusion coefficient of FA mortar increases gradually. The addition of 15% LS leads to an increase of 40.50% in the chloride diffusion coefficient compared to the mortar with 60% FA. This indicates that the addition of any proportion of LS will reduce the chloride ion erosion resistance of FA mortar. This is mainly because, for the mortar with 60% FA, the structure is extremely loose due to fewer hydration products. When LS further replaces cement, hydration products will be further reduced, which will make the internal structure of mortar looser, leading to a reduction in chloride ion resistance of FA mortar.

Figure 5b displays the relationship between the chloride diffusion coefficient and compressive strength. The chloride diffusion coefficient has a negative correlation with compressive strength; that is, the sample with higher compressive strength has a lower chloride diffusion coefficient. Meanwhile, the R^2^ of the fitted equation, which is greater than 0.95, verifies the accuracy of this negative correlation relationship.

### 3.5. XRD

**Figure 6:**
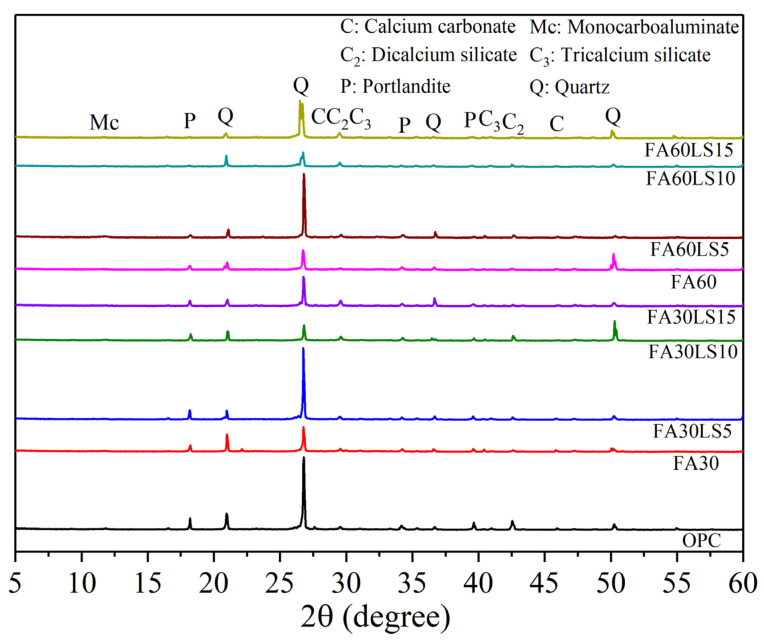
XRD patterns of FA mortars containing different dosages of LS.

Figure 6 displays XRD patterns of FA mortars containing different dosages of LS cured for 180 d. It can be observed that the mortar without FA and LS exhibits the strongest peak of Ca(OH)_2_, which means that it obtains the most C-S-H gels. This also enables the OPC group to achieve a compressive strength that is much higher than that of all the other groups at 180 d. With an increase in FA, the peak of Ca(OH)_2_ becomes weaker, which is mainly due to the replacement of cement by FA and the reaction of FA with Ca(OH)_2_ due to the volcanic ash effect. The weakening of the Ca(OH)_2_ peak indicates a decrease in the amount of C-S-H, which results in the compressive strength of mortar with FA being lower than that of mortar without FA. For the mortar containing 30% FA, the peak of Ca(OH)_2_ is enhanced by adding 5% LS compared with that without LS. This is mainly due to that fine LS particles can provide nucleation sites for cement hydration to promote the generation of more hydration products, and the chemical effect of LS can produce additional hydration products of monocarbonate and hemicarboaluminate [47]. The peak enhancement of Ca(OH)_2_ indicates an increase in C-S-H. This also explains why the compressive strength of FA mortar with 5% LS added is greater than that of FA mortar without LS added. However, when the addition amount of LS is 10% and 15%, the increase in Ca(OH)_2_ caused by the nucleation effect of LS is less than the reduction in Ca(OH)_2_ caused by the replacement of cement by LS and ultimately leads to the reduction in Ca(OH)_2_. This also means that a large amount of LS will lead to a reduction in C-S-H, which is the component that determines the strength of mortar. Therefore, the incorporation of 10% and 15% LS eventually leads to some reduction in the compressive strength of FA mortar. Furthermore, for the mortar containing 60% FA, the peak of Ca(OH)_2_ decreases with an increase in LS. This is mainly because the replacement of cement by 60% FA has resulted in a significant reduction in hydration products, and the replacement of cement by LS will result in a further reduction in hydration products. Moreover, due to the reduction in cement particles, the nucleation effect of LS on the promotion of hydration becomes very weak, and it is almost impossible to increase the hydration products. This also leads to the fact that, when the FA dosage is 60%, any LS dosage cannot improve the compressive strength of FA mortar.

### 3.6. SEM

**Figure 7:**
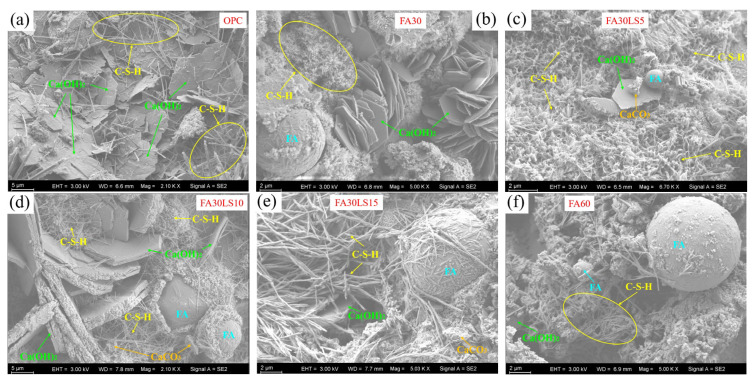

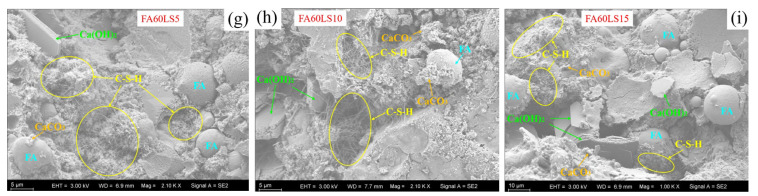
Micromorphology of FA mortars containing different dosages of LS cured for 180 d. (**a**) OPC; (**b**) FA30; (**c**) FA30LS5; (**d**) FA30LS10; (**e**) FA30LS15; (**f**) FA60; (**g**) FA60LS5; (**h**) FA60LS10; (**i**) FA60LS15.

Figure 7 presents the micromorphology of FA mortars containing different dosages of LS cured for 180 d. For the mortar without FA and LS (Figure 7a), a large number of fibrous C-S-H gels and plate Ca(OH)_2_ can be observed, and the two hydration products interlace with each other, forming a dense internal structure. This enables the mortar without FA and LS to have the highest compressive strength. Furthermore, for the mortars containing 30% FA and different dosages of LS (Figure 7b–e), the spherical FA and large amounts of C-S-H and Ca(OH)_2_ are clearly visible. Meanwhile, the fine LS particles are filled in the interstice of FA mortars. However, it can be observed that, since FA occupies a large portion of the matrix and FA has not fully hydrated, the compressive strength of FA mortar, regardless of whether LS is added or not, is always lower than that of mortar without FA and LS. Furthermore, it should be pointed out that, when the LS content is 5% (Figure 7c), C-S-H in FA mortar is flocculent, and FA, Ca(OH)_2_, and CaCO_3_ are cross-stacked in C-S-H, making FA mortar have the densest internal structure. This results in the compressive strength of FA mortar with 5% LS added being larger than that of FA mortar without LS added. However, when the LS content is 10% and 15% (Figure 7d,e), although FA, Ca(OH)_2_, and CaCO_3_ are also cross-deposited in C-S-H, the internal structure of FA mortar becomes relatively loose. This results in the compressive strength of FA mortar with the addition of LS being lower than that of FA mortar without the addition of LS, and the greater the amount of LS added, the lower the compressive strength of FA mortar becomes. In addition, for the mortars containing 60% FA and different dosages of LS (Figure 7f–i), the decrease in hydration products C-S-H and Ca(OH)_2_ can be significantly observed. Moreover, with an increase in LS, the hydration products, C-S-H gels, and Ca(OH)_2_ in FA mortars are further reduced. Meanwhile, it can still be observed that fine particles of LS can be filled between FA particles and hydration products, but the increase in LS does not significantly increase hydration products, resulting in the cementitious ability and loose structure of the mortars containing 60% FA that cannot be improved. This also leads to the fact that the compressive strength of mortar with 60% FA added is much lower than that of mortar with 30% FA added. Moreover, the addition of any LS content does not enhance the compressive strength of FA mortar, and as the amount of LS added increases, the compressive strength of FA mortar gradually decreases.

### 3.7. TG

**Figure 8:**
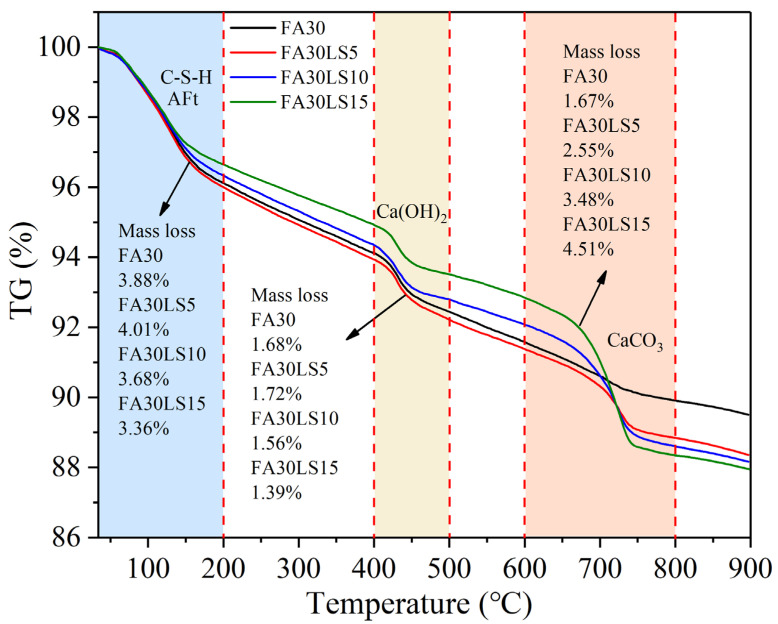
TG curves of FA mortars containing different dosages of LS.

Figure 8 presents TG curves of FA mortars containing different dosages of LS cured for 180 d. Overall, TG curves show three distinct sudden drops, corresponding to three different temperature ranges: 35~200 °C, 400~500 °C, and 600~800 °C. The first stage, ranging from 35 °C to 200 °C, mainly corresponds to the loss of the bound water in C-S-H and AFt [48]. The second stage, ranging from 400 to 500 °C [49], mainly corresponds to the loss of water after the decomposition of Ca(OH)_2_. The third stage, ranging from 600 to 800 °C, mainly corresponds to the release of CO_2_ after the decomposition of CaCO_3_ [50].

From the mass loss of the first and second stages, it can be seen that the addition of 5% LS can increase mass losses of C-S-H, AFt, and Ca(OH)_2_, indicating that 5% LS can promote the hydration of FA mortar to generate more hydration products. This is mainly because the nucleation effect of LS can facilitate the hydration of more cement particles [51], which is the reason 5% LS can increase the compressive strength and durability of FA mortar. However, when the LS additions reach 10% and 15%, the mass losses of C-S-H, AFt, and Ca(OH)_2_ decrease gradually, indicating that 10% and 15% LS will result in a reduction in hydration products of FA mortar. This is due to the increase in the amount of hydration products resulting from the nucleation effect of LS that cannot compensate for the reduction in hydration products caused by excessive substitution of LS for cement. Therefore, when 10% and 15% LS are added to FA mortar, the enhancing effect of LS on the compressive strength is much weaker than its weakening effect, ultimately leading to a decrease in the compressive strength of FA mortar. From the quality loss data of the third stage, the continuous increase in LS dosage will lead to a continuous increase in the quality loss of CaCO_3_. This is mainly due to the fact that LS only has weak reactivity, and most LS cannot participate in a hydration reaction. As a result, the mass loss of CaCO_3_ increases with an increase in LS.

### 3.8. MIP

**Figure 9:**
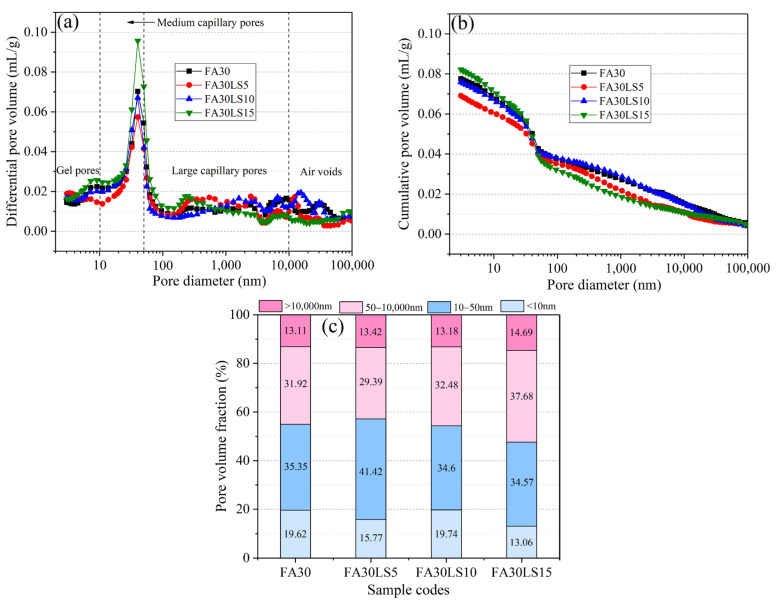
Pore compositions of FA mortars containing different dosages of LS: (**a**) differential pore volume; (**b**) cumulative pore volume; (**c**) pore volume fraction.

Figure 9 presents the FA mortars containing different dosages of LS cured for 180 d. From Figure 9a, it can be observed that the pore diameters of FA mortars containing LS are divided into four types: gel pores smaller than 10 nm, medium capillary pores of 10~50 nm, large capillary pores of 50~10,000 nm, and air voids larger than 10,000 nm [52]. The pore diameters of FA mortars containing LS are mainly distributed in medium capillary pores and large capillary pores. The cumulative pore volume of FA mortars with LS is presented in Figure 9b. It can be observed that FA30LS5 obtains the smallest cumulative pore volume, FA30LS15 obtains the largest cumulative pore volume, and FA30 and FA30LS10 obtain very close cumulative pore volumes. Furthermore, Figure 9c displays the pore volume fraction for different types of pore diameters of FA mortars containing different dosages of LS. Previous scholars have found that pores with a diameter greater than 50 nm will cause greater damage to the strength and durability of cement-based materials [21,49]. Hence, it can be found that, after adding 5% LS, FA mortar can obtain the minimum total amount of large capillary pores and air pores. However, when 10% and 15% LS are added, the total amount of large capillary pores and air pores of FA mortar gradually increases. Meanwhile, the total porosity and average pore size of FA mortars containing different dosages of LS are listed in Table 5. It can be found that the FA mortar containing 5% LS obtains a smaller total porosity and average pore size than that of FA mortar without LS. Moreover, with the LS dosages increasing to 10% and 15%, the total porosity and average pore size of FA mortars increase gradually compared with that of FA mortar without LS. The results aforementioned above indicate that a small amount of LS can compact the internal structure of FA mortar, while a higher amount of LS will lead to a loose internal structure of FA mortar. This is mainly because the filling effect of fine LS can fill the pores between hydration products and aggregates, and hydration products generated by the activity effect of LS can jointly enhance the compactness of FA mortar [53]. However, when the LS content is too large, the filling effect and chemical activity effect of LS are not enough to make up for the loose internal structure of FA mortar caused by the reduction in hydration products caused by LS replacing cement.

### 3.9. Relationship Between Mechanical Strength and Environmental Effect

**Figure 10:**
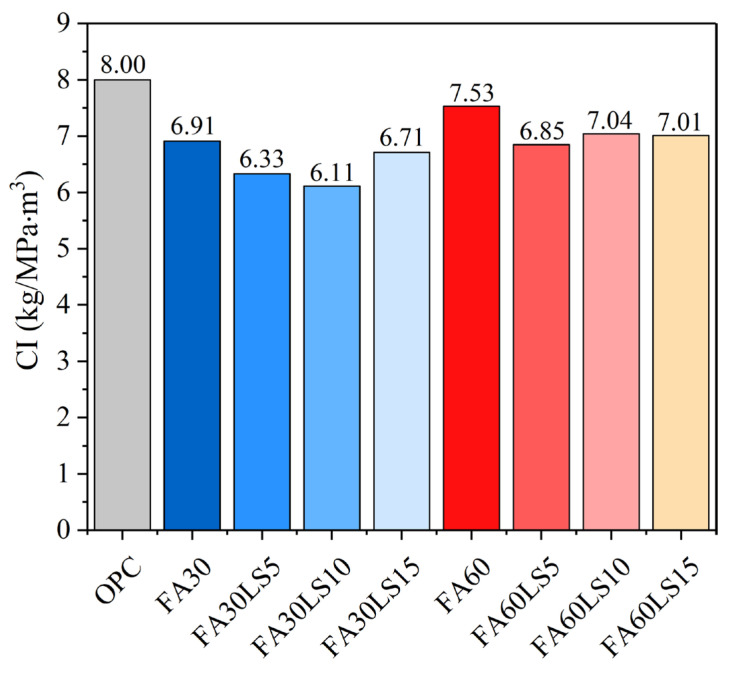
CI values of mortars with different dosages of FA and LS.

Figure 10 presents the CI values of mortars containing different dosages of FA and LS at 180 d. It can be seen that, when FA and LS are added to mortar, the CI values all decrease to varying degrees, indicating that the CO_2_ emissions per unit of compressive strength have decreased. Therefore, the addition of FA and LS can improve the environmental friendliness of the mortar. Furthermore, for the mortar containing 30% FA, with an increase in LS dosage, the CI value first decreases and then increases, and it reaches the minimum when the LS dosage is 10%. This demonstrates that, in a mortar with 30% FA, adding 10% LS can result in the lowest unit CO_2_ emission, and it has the best environmental friendliness. Moreover, for the mortar containing 60% FA, adding different dosages of LS to FA mortar can all reduce the CI values, and when LS dosage is 5%, the CI value of FA mortar reaches the minimum. This reveals that, in a mortar with 60% FA, adding 5% LS can result in the lowest unit CO_2_ emission, and it has the best environmental friendliness.

## 4. Conclusions

This study investigated the mechanical strength and resistance to freeze–thaw cycles and chloride ions of FA mortar mixed with LS cured under a low temperature of 5 °C. Through tests on the compressive strength and resistance to freeze–thaw cycles and chloride ions, as well as characterization of hydration products and microstructure, the main conclusions are drawn:

(1) For the mortar containing 30% FA, the addition of 5% and 10% LS can improve the compressive strength of FA mortar at 3 d and 28 d, and only 5% LS can improve the strength of FA mortar at 90 d and 180 d. Adding 5% LS can increase the compressive strength of FA mortar by 14.31% at 3 d and 1.63% at 180 d. However, 15% LS will reduce the compressive strength of FA mortar from 3 d to 180 d. For the mortar containing 60% LS, adding LS does not increase the strength of FA mortar from 3 d to 180 d.
(2) For the mortar containing 30% FA, the addition of 5% LS results in a 0.58% reduction in the mass loss rate and an increase of 2.98% in RDM compared to FA mortar after 100 freeze–thaw cycles. However, the addition of 10% and 15% LS will lead to an increase in mass loss rates and a decrease in RDM of FA mortars. For the mortar containing 60% LS, with the addition of LS from 5% to 15%, the mass loss rate increases sharply, while the RDM decreases sharply.
(3) The mortar without FA and LS can obtain the most C-S-H gels. For the mortar containing 30% FA, the addition of 5% LS can significantly promote the hydration products of C-S-H, while 10% and 15% will gradually decrease the hydration products of C-S-H. For the mortar containing 60% FA, the increasing dosage of LS will lead to the gradual decrease in C-S-H gels.
(4) The FA mortar containing 5% LS and 15% LS can result in the smallest cumulative pore volume and the largest cumulative pore volume, respectively. Meanwhile, the FA mortar containing 5% LS can obtain the minimum total amount of large capillary pores and air pores. However, 10% and 15% LS will lead to the total amount of large capillary pores and air pores of FA mortar gradually increasing.
(5) The addition of FA and LS can improve the environmental friendliness of mortar. For the mortar containing 30% FA, with an increase in LS dosage, the CI value first decreases and then increases, and it reaches the minimum when the LS dosage is 10%. For the mortar containing 60% FA, adding different dosages of LS to FA mortar can all reduce the CI values, and when LS dosage is 5%, the CI value of FA mortar reaches the minimum.

## Figures and Tables

**Table 1:** Chemical compositions of OPC, FA, and LS (wt%).

Samples	CaO	SiO_2_	Al_2_O_3_	Fe_2_O_3_	MgO	SO_3_	K_2_O	LOI
OPC	62.58	20.39	5.43	3.76	1.73	2.28	0.78	3.05
FA	2.47	48.92	36.86	6.79	0.73	0.58	1.07	2.58
LS	56.34	0.37	0.19	0.18	1.69	–	–	41.23

**Table 2:** Mix proportions of mortars with different dosages of FA and LS (kg/m^3^).

Samples	w/b	Water	Cement	FA	LS	Sand	SP
OPC	0.35	194	554	0	0	1662	2.77
FA30	0.35	194	387.8	166.2	0	1662	2.77
FA30LS5	0.35	194	360.1	166.2	27.7	1662	2.77
FA30LS10	0.35	194	332.4	166.2	55.4	1662	2.77
FA30LS15	0.35	194	304.7	166.2	83.1	1662	2.77
FA60	0.35	194	221.6	332.4	0	1662	2.77
FA60LS5	0.35	194	193.9	332.4	27.7	1662	2.77
FA60LS10	0.35	194	166.2	332.4	55.4	1662	2.77
FA60LS15	0.35	194	138.5	332.4	83.1	1662	2.77

**Table 3:** The raw materials used to prepare mortar containing FA and LS.

Items	$e-CO_{2}$ (kg/m^3^)	References
Cement	0.83	[34,35]
FA	0.009	[33,34]
LS	0.017	[33,36]
Sand	0.001	[33,36]
SP	0.72	[33,37]
Water	0.0003	[33,38]

**Table 4:** Correlation coefficients of the fitting curves between compressive strength loss percentages and freeze–thaw cycles.

Samples	A	B	C	R^2^
OPC	9.405	68.363	−9.641	0.9993
FA30	8.180	62.531	−8.269	0.9998
FA30LS5	6.969	62.255	−7.370	0.9981
FA30LS10	8.551	63.747	−8.646	0.9999
FA30LS15	8.926	64.640	−9.012	0.9999
FA60	37.771	93.262	−38.686	0.9986
FA60LS5	44.841	102.870	−45.671	0.9982
FA60LS10	51.232	110.620	−51.879	0.9993
FA60LS15	46.467	96.882	−46.645	0.9999

**Table 5:** Total porosity and average pore size of FA mortars containing different dosages of LS.

Samples	Total Porosity (%)	Average Pore Size (nm)
FA30	15.75	26.01
FA30LS5	14.52	24.21
FA30LS10	16.12	26.95
FA30LS15	17.01	27.61

## Data Availability

The original contributions presented in this study are included in the article. Further inquiries can be directed to the corresponding author.
